# Supplementary material for: Epstein–Barr virus nuclear antigen 2 extensively rewires the human chromatin landscape at autoimmune risk loci
Source: Genome Res. 2021 Dec;31(12):2185–98. doi: 10.1101/gr.264705.120 (PMC8647835; doi:10.1101/gr.264705.120)
Supplement: Supplemental Material [file supp_31_12_2185__DC1.html]

Epstein–Barr virus nuclear antigen 2 extensively rewires the human chromatin landscape at autoimmune risk loci — Supplemental Material 

# Epstein–Barr virus nuclear antigen 2 extensively rewires the human chromatin landscape at autoimmune risk loci

## Supplemental Material

- Supplemental\_Methods\_THIRD\_REVISION.docx
- Supplemental\_Fig\_S1.pdf
- Supplemental\_Fig\_S2.pdf
- Supplemental\_Fig\_S3.pdf
- Supplemental\_Fig\_S4.pdf
- Supplemental\_Fig\_S5.pdf
- Supplemental\_Code.zip
- Supplemental\_Table\_S1.xlsx
- Supplemental\_Table\_S2.xlsx
- Supplemental\_Table\_S3.xlsx
- Supplemental\_Table\_S4.xlsx
- Supplemental\_Table\_S5\_THIRD\_REVISION.xlsx
- Supplemental\_Table\_S6\_THIRD\_REVISION.xlsx
- Supplemental\_Table\_S7.xlsx
- Supplemental\_Table\_S8.xlsx
- Supplemental\_Table\_S9.xlsx
- Supplemental\_Table\_S10.xlsx
- Supplemental\_Table\_S11.xlsx
- Supplemental\_Table\_S12.xlsx
- Supplemental\_Table\_S13.xlsx
- Supplemental\_Table\_S14.xlsx
- Supplemental\_Table\_S15.xlsx
- Supplemental\_Table\_S16.xlsx
