# Supplementary material for: Epstein–Barr virus nuclear antigen 2 extensively rewires the human chromatin landscape at autoimmune risk loci
Source: Genome Res. 2021 Dec;31(12):2185–98. doi: 10.1101/gr.264705.120 (PMC8647835; doi:10.1101/gr.264705.120)
Supplement: Supplemental Material [file supp_gr.264705.120_Supplemental_Methods_THIRD_REVISION.docx]

**Supplemental Methods**

**RNA-seq**

Total RNA was extracted using the mirVANA Isolation Kit (Ambion) from Ramos B cells in each of the three conditions: uninfected Ramos (uninfected), EBNA2-positive EBV (B95.8)-infected Ramos (EBV^EBNA2+^), and EBNA2-negative EBV (P3HR-1)-infected Ramos (EBV^EBNA2-^). RNA was extracted at the same time point for every condition. RNA was depleted of ribosomal RNA using the Ribo-Zero rRNA removal kit and sequenced at Cincinnati Children’s Hospital Medical Center (CCHMC) DNA Sequencing and Genotyping Core Facility. Using the tool FastQC (version: 0.11.2) (http://www.bioinformatics.babraham.ac.uk/projects/fastqc), all data were confirmed to pass all quality control checks, except for adapter sequence contents, which were removed using cutadapt (Martin 2011) (trimgalore version: 0.4.2) (https://journal.embnet.org/index.php/embnetjournal/article/view/200). RNA-seq reads were aligned to the hg19 (GrCh37) genome build (NCBI) using Spliced Transcripts Alignment to a Reference (STAR, version: 020201) (https://doi.org/10.1093/bioinformatics/bts635). The featureCounts function (Rsubread version: 3.5.3) was used to count reads (Liao et al. 2014) and DESeq2 (version 3.5.3) was used to perform differential gene expression (Love et al. 2014). DESeq2 results were filtered to only include genes with fragments per kilobase per million (FPKM) values greater than one in at least one condition. Differential expression was calculated with thresholds of a greater than 1.5-fold change and an adjusted *p*-value threshold of less than 0.05 (*p*-values were adjusted by Benjamini and Hochberg false discovery rate (FDR)). Gene set enrichment analysis was performed using ToppFun (Chen et al. 2009).

**ChIP-seq**

Ramos and GM12878 cells were crosslinked and nuclei were sonicated as described previously (Lu et al. 2015). Cells were incubated in crosslinking solution (1% formaldehyde, 5 mM HEPES [pH 8.0], 10 mM sodium chloride, 0.1 mM EDTA, and 0.05 mM EGTA in RPMI medium 1640 with 10% FBS) and placed on a tube rotator at room temperature for 10 min. To stop the crosslinking, glycine was added to a final concentration of 0.125 M and tubes were placed back on the rotator at room temperature for 5 min. Cells were washed twice with ice-cold PBS, resuspended in lysis buffer 1 (50 mM HEPES [pH 7.5], 140 mM NaCl, 1 mM EDTA, 10% glycerol, 0.25% Triton X-100, and 0.5% NP-40), and incubated for 10 min on ice. Nuclei were harvested after centrifugation at 5,000 rpm for 10 min, resuspended in lysis buffer 2 (10 mM Tris-HCl [pH 8.0], 1 mM EDTA, 200 mM NaCl, and 0.5 mM EGTA), and incubated at room temperature for 10 min. Protease and phosphatase inhibitors were added to both lysis buffers. Nuclei were then resuspended in sonication buffer (10 mM Tris [pH 8.0], 1 mM EDTA, and 0.1% SDS). A S220 focused ultrasonicator (COVARIS) was used to shear chromatin (150- to 500-bp fragments) with 10% duty cycle, 175 peak power, and 200 bursts per cycle for 7 min. A portion of the sonicated chromatin was run on an agarose gel to verify fragment sizes. Sheared chromatin was precleared with 20 μL Dynabeads Protein G (Life Technologies) at 4 °C for 40 min.

Immunoprecipitation of EBNA2-chromatin complexes was performed with an SX-8X IP-STAR compact automated system (Diagenode). Beads conjugated to antibodies against EBNA2 (Abcam; ab90543) were incubated with precleared chromatin at 4°C for 8 hours. The beads were then washed sequentially with wash buffer 1 (10 mM Tris-HCl [pH 7.5], 150 mM NaCl, 1 mM EDTA, 0.1% SDS, 0.1% NaDOC, and 1% Triton X-100), wash buffer 2 (10 mM Tris-HCl [pH 7.6], 400 mM NaCl, 1 mM EDTA, 0.1% SDS, 0.1% NaDOC, and 1% Triton X-100), wash buffer 3 (10 mM Tris-HCl [pH 8.0], 250 mM LiCl, 1 mM EDTA, 0.5% NaDOC, and 0.5% NP-40), and wash buffer 4 (10 mM Tris-HCl [pH 8.0], 1 mM EDTA, and 0.2% Triton X-100). Finally, the beads were resuspended in 10 mM Tris-HCl (pH 7.5) and used to prepare libraries via ChIPmentation (Schmidl et al. 2015).

Published EBNA2 ChIP-seq data sets used in this study are available in the NCBI Short Read Archive (SRA; https://www.ncbi.nlm.nih.gov/sra) under accession numbers SRX1530787 (GM12878 cells), SRX092451 (IB4 cells), and SRX290877 (Mutu cells).

**ATAC-seq**

ATAC-seq was performed using standard conditions. Transposase Tn5 with adapter sequences was used to cut accessible DNA. These accessible DNA with adaptor sequences were isolated, then libraries were prepared for uninfected, EBV^EBNA2-^, EBV^EBNA+^ Ramos cells using a standard protocol (Buenrostro et al. 2015)*.* Paired-end sequencing was performed at the CCHMC DNA Sequencing and Genotyping Core Facility.

**HiChIP-seq**

HiChIP-seq libraries were prepared following Mumbach *et al.* (Mumbach et al. 2016). Cells were cross-linked with 1% formaldehyde and lysed with Hi-C lysis buffer. Cross-linked DNA was digested by MboI. DNA ends were filled with biotin-dATP and dCTP, dTTP, dGTP and then ligated by T4 DNA ligase. After ligation, DNA was sonicated using a Covaris M220. Fragmented DNA was then diluted 10 times with ChIP dilution buffer and protein-DNA complexes were captured using an H3K27ac antibody (Abcam AB4729). Next, protein-DNA complexes were further captured by protein A beads and eluted. DNA was purified after reverse crosslinking. Biotin dATP-labeled DNA was captured with Streptavidin C-1 beads and PCR amplified using Phusion HF (New England Biosciences) and 1 μL of each Nextera forward primer (Ad1_noMX) and Nextera reverse primer (Ad2.X). The libraries were sequenced using Illumina NextSeq (2x75bp).

**References**

Buenrostro JD, Wu B, Chang HY, Greenleaf WJ. 2015. ATAC-seq: A Method for Assaying Chromatin Accessibility Genome-Wide. *Curr Protoc Mol Biol* **109**: 21 29 21-29.

Chen J, Bardes EE, Aronow BJ, Jegga AG. 2009. ToppGene Suite for gene list enrichment analysis and candidate gene prioritization. *Nucleic Acids Res* **37**: W305-311.

Liao Y, Smyth GK, Shi W. 2014. featureCounts: an efficient general purpose program for assigning sequence reads to genomic features. *Bioinformatics* **30**: 923-930.

Love MI, Huber W, Anders S. 2014. Moderated estimation of fold change and dispersion for RNA-seq data with DESeq2. *Genome Biol* **15**: 550.

Lu X, Zoller EE, Weirauch MT, Wu Z, Namjou B, Williams AH, Ziegler JT, Comeau ME, Marion MC, Glenn SB et al. 2015. Lupus Risk Variant Increases pSTAT1 Binding and Decreases ETS1 Expression. *Am J Hum Genet* **96**: 731-739.

Martin M. 2011. Cutadapt removes adapter sequences from high-throughput sequencing reads. *2011* **17**: 3.

Mumbach MR, Rubin AJ, Flynn RA, Dai C, Khavari PA, Greenleaf WJ, Chang HY. 2016. HiChIP: efficient and sensitive analysis of protein-directed genome architecture. *Nat Methods* **13**: 919-922.

Schmidl C, Rendeiro AF, Sheffield NC, Bock C. 2015. ChIPmentation: fast, robust, low-input ChIP-seq for histones and transcription factors. *Nature methods* **12**: 963-965.
