# Supplementary material for: Epstein–Barr virus nuclear antigen 2 extensively rewires the human chromatin landscape at autoimmune risk loci
Source: Genome Res. 2021 Dec;31(12):2185–98. doi: 10.1101/gr.264705.120 (PMC8647835; doi:10.1101/gr.264705.120)
Supplement: Supplemental Material [file supp_gr.264705.120_Supplemental_Fig_S1.pdf]

A

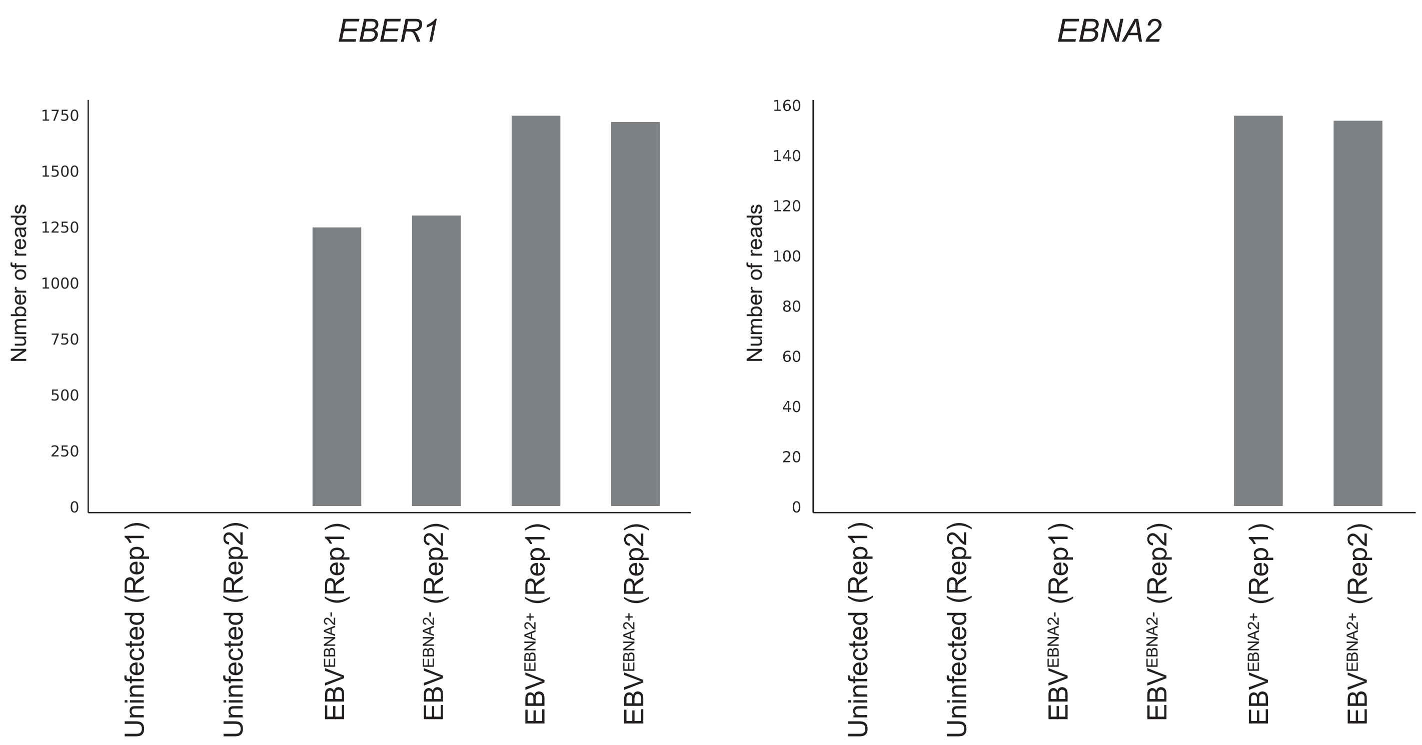

B

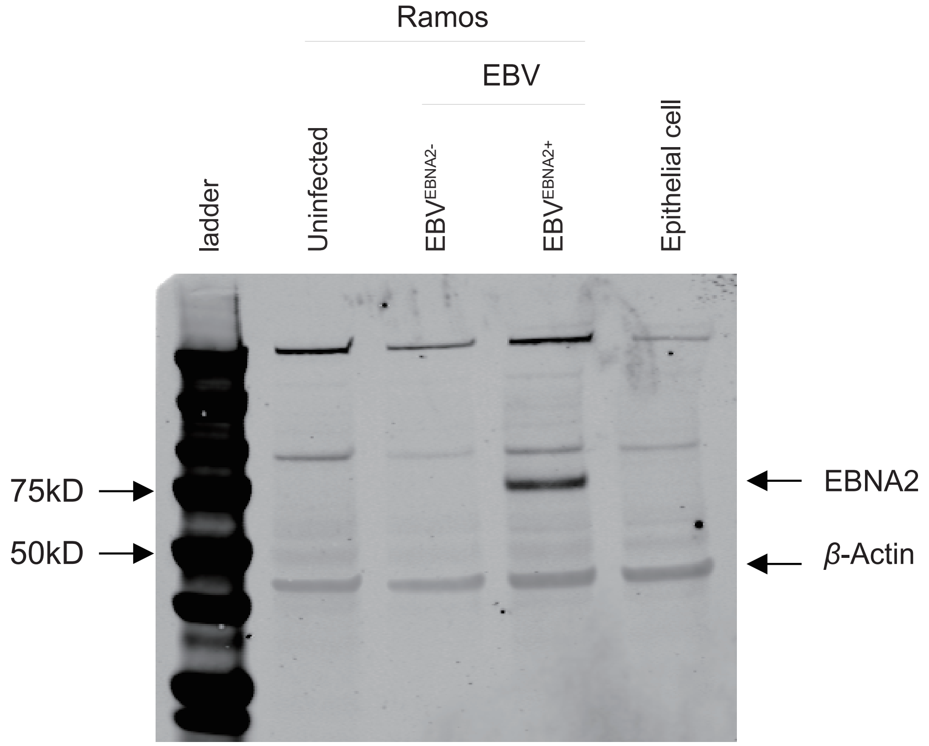

Supplemental Figure 1. Expression of EBV genes in uninfected, EBV^{EBNA2-}, and EBV^{EBNA2+} Ramos cells. (*A*) Gene expression (read count) of *EBER1* and *EBNA2* among infection types. (*B*) Western blot showing differential EBNA2 expression among infection types.
