# Supplementary material for: Epstein–Barr virus nuclear antigen 2 extensively rewires the human chromatin landscape at autoimmune risk loci
Source: Genome Res. 2021 Dec;31(12):2185–98. doi: 10.1101/gr.264705.120 (PMC8647835; doi:10.1101/gr.264705.120)
Supplement: Supplemental Material [file supp_gr.264705.120_Supplemental_Fig_S2.pdf]

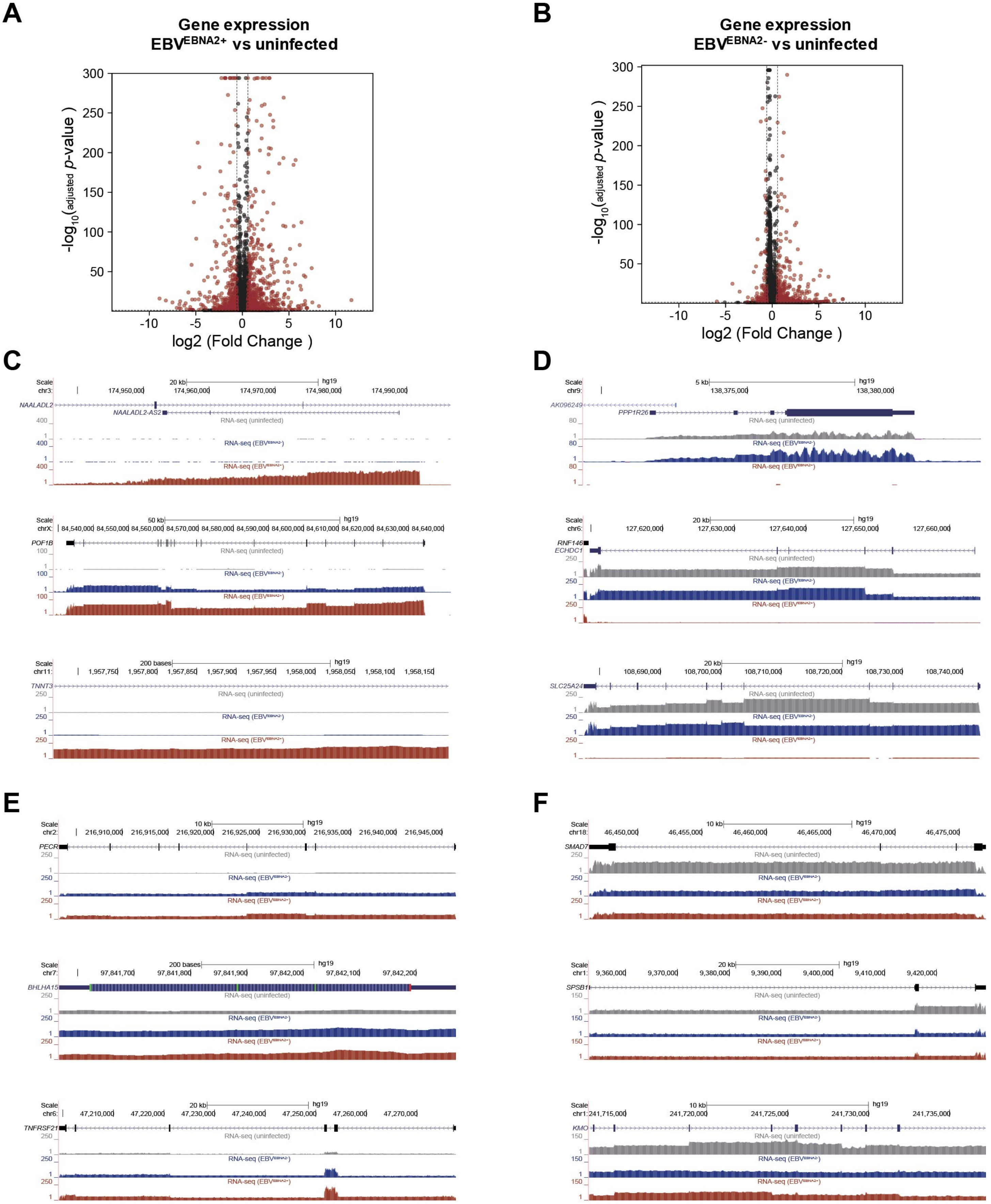

Supplemental Figure 2. EBNA2-dependent human gene expression in Ramos B cells. (*A*,*B*). Volcano plots showing differential human gene expression upon infection with EBV^{EBNA2+} (*A*) and EBV^{EBNA2-} (*B*) virus. (*C*,*D*) Examples of EBNA2-dependent differentially expressed genes in the UCSC Genome Browser (hg19) showing up-regulated genes (*C*) and down-regulated genes(*D*) . (*E*,*F*) UCSC Genome Browser (hg19) depicting the EBV-dependent EBNA2-independent up-regulated genes (*E*) and down-regulated genes (*F*).
