# Supplementary material for: Epstein–Barr virus nuclear antigen 2 extensively rewires the human chromatin landscape at autoimmune risk loci
Source: Genome Res. 2021 Dec;31(12):2185–98. doi: 10.1101/gr.264705.120 (PMC8647835; doi:10.1101/gr.264705.120)
Supplement: Supplemental Material [file supp_gr.264705.120_Supplemental_Fig_S3.pdf]

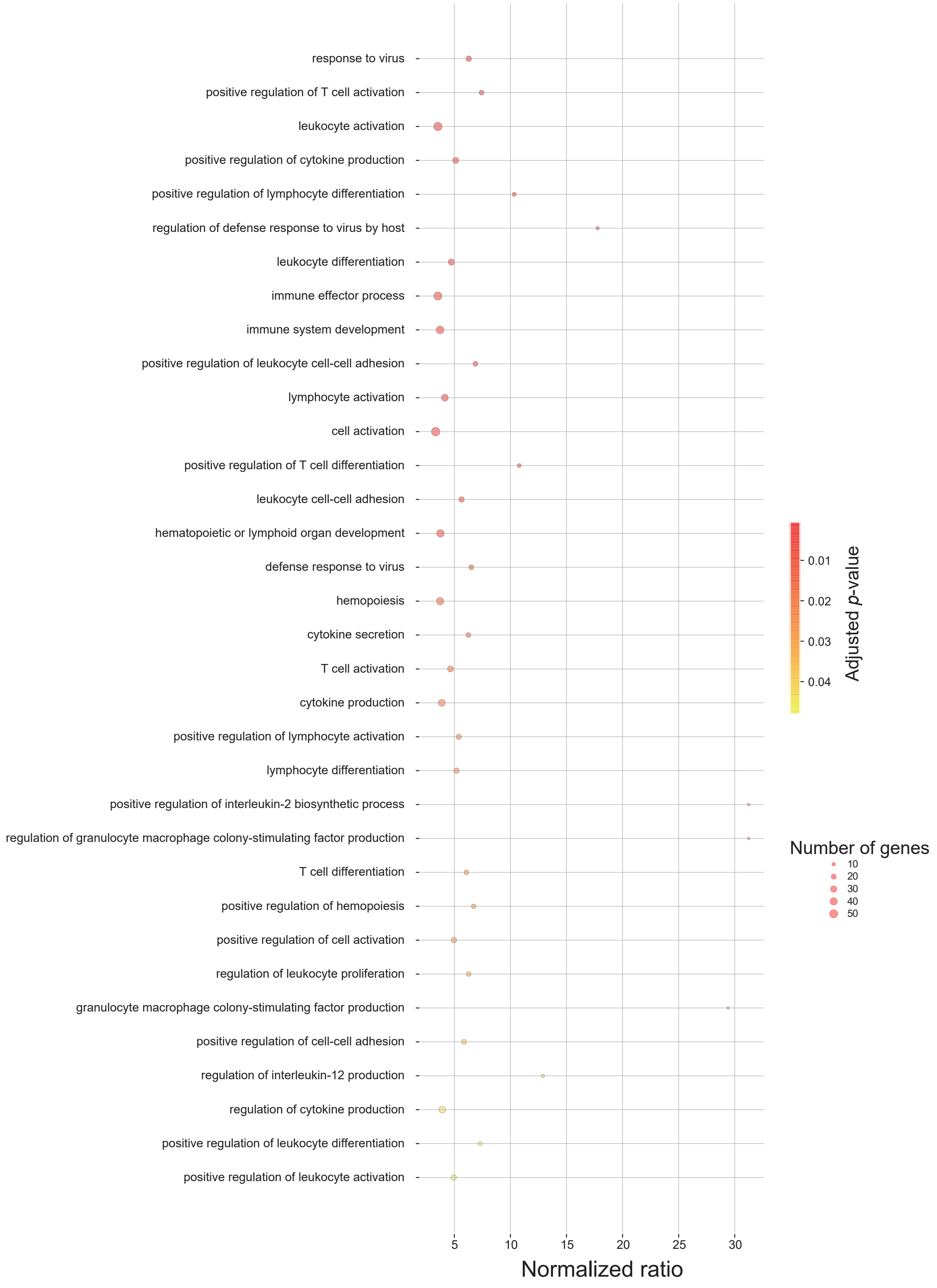

Supplemental Figure 3. Functional enrichment analysis for EBNA2-dependent differentially expressed genes. Gene set enrichment analyses of EBNA2-dependent differentially expressed genes for GO Biological Process. The X-axis indicates the normalized ratio of the number of input genes to the total number of annotated genes times 100. The Y-axis is sorted by significance (i.e., lower p-values are at the top of the plot).Analysis was performed using ToppFun from ToppGene Suite (https://toppgene.cchmc.org/enrichment.jsp). Bonferroni correction was used to adjust the *p*-values.
