# Supplementary material for: Epstein–Barr virus nuclear antigen 2 extensively rewires the human chromatin landscape at autoimmune risk loci
Source: Genome Res. 2021 Dec;31(12):2185–98. doi: 10.1101/gr.264705.120 (PMC8647835; doi:10.1101/gr.264705.120)
Supplement: Supplemental Material [file supp_gr.264705.120_Supplemental_Fig_S5.pdf]

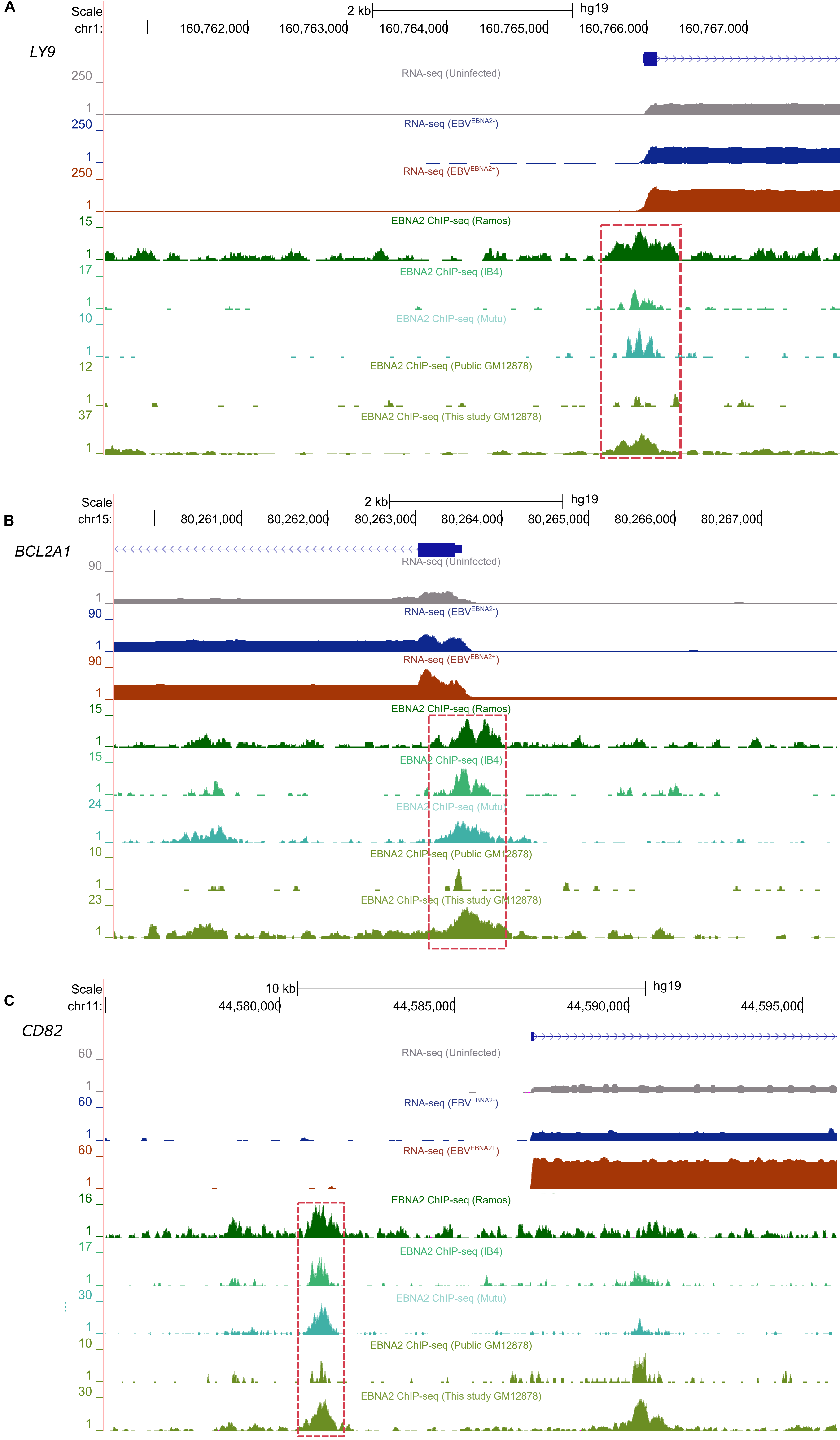

Supplemental Figure 5. EBNA2 binding at the promoter of EBNA2-dependent up-regulated genes (A-C). UCSC Genome Browser shots (hg19) for the LY9 (A), BCL21A (B), and CD82 (C) promoter regions. EBNA2 ChIP-seq peaks are boxed in green.
